# Intraventricular metastatic melanoma: A case report and review of the literature

**DOI:** 10.1002/ccr3.2983

**Published:** 2020-07-03

**Authors:** Joshua D. Bernstock, Gustavo Chagoya, Galal A. Elsayed, Brandon M. Fox, Nabiel Mir, Saksham Gupta, Melissa Chua, Travis J. Atchley, Mina Lobbous, Houman Sotoudeh, James Hackney, Gregory K. Friedman, Mark R. Harrigan

**Affiliations:** ^1^ Department of Neurosurgery Brigham and Women's Hospital Harvard Medical School Boston MA USA; ^2^ Department of Neurosurgery The University of Alabama at Birmingham Birmingham AL USA; ^3^ Medical Scientist Training Program The University of Alabama at Birmingham Birmingham AL USA; ^4^ Department of Internal Medicine The University of Alabama at Birmingham Birmingham AL USA; ^5^ Division of Neuro‐Oncology Department of Neurology University of Alabama at Birmingham Birmingham AL USA; ^6^ Department of Neuroradiology University of Alabama at Birmingham Birmingham AL USA; ^7^ Department of Pathology University of Alabama at Birmingham Birmingham AL USA; ^8^ Division of Pediatric Hematology and Oncology Department of Pediatrics University of Alabama at Birmingham Birmingham AL USA

**Keywords:** central nervous system metastasis, intraventricular metastasis, melanoma, metastatic melanoma

## Abstract

Intraventricular melanoma is a very rare and highly malignant disease. Safe resection is the mainstay of treatment, but no standard guidelines exist for adjuvant therapy. Early histologic and molecular diagnosis is key for improved survival.

## Graphical abstract

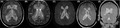

## 1 INTRODUCTION

Stage IV melanoma involving the central nervous system (CNS) represents approximately 10% of all brain metastases among adults and is the third most common cancer to metastasize to the brain, behind only primary cancers of the lung and breast.^1^, ^2^ The pathophysiology of melanoma CNS metastasis includes invasion, intravasation, dissemination, arrest, extravasation, angiogenesis, and proliferation. Interaction with the blood‐brain barrier is mediated by arrest, the physical obstruction of brain capillaries with tumor cells and enhanced cellular adhesion. Recent literature suggests phosphoinositide 3‐kinases (PI3K) regulate mediators, such as Rac, are responsible for increased adhesion to brain endothelium by altering tumor cell polarization through actin ultrastructure.^3^ Melanoma cells have demonstrated increased adhesion to brain endothelium as compared to breast cancer cells in animal models.^4^ PI3K inhibitors have demonstrated some in vitro success in preventing adhesion to brain endothelium but are still in early stages of development.^3^ The clinical significance of CNS involvement in melanoma is highlighted by a separate M category, M1d, in the eighth edition of the American Joint Committee on Cancer (AJCC) TNM staging system for melanoma.^5^

Of note, the majority of cases of melanoma involving the CNS are typically intraparenchymal, leptomeningeal, or dural based. Intraventricular (IVT) lesions represent a rare site of CNS involvement in melanoma.^6^ IVT metastasis is an extremely uncommon clinical entity irrespective of primary cancer site, but interestingly, one study reported melanoma represented 14% of 35 cases of IVT metastasis, second only to renal cell carcinoma.^7^ Herein, we present a case of metastatic melanoma involving the ventricular spaces and review the literature surrounding IVT melanoma.

## 2 CASE

**FIGURE 1:**
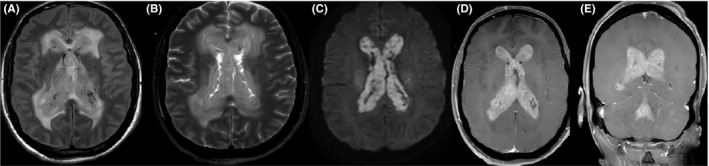
Intraventricular melanoma metastasis. A and B, axial FLAIR and T2 sequences demonstrate mild dilation of the ventricular system with near complete obliteration of the lateral ventricles by a heterogeneous and mainly hyperintense soft tissue mass. In addition, there is significant periventricular FLAIR/T2 signal hyperintensity consistent with subependymal interstitial edema. C, Axial diffusion‐weighted imaging (DWI) demonstrates a soft tissue mass within the ventricular system, which shows marked diffusion restriction. (D) Axial and (E) coronal T1 postcontrast sequences show near‐complete obliteration of the ventricular system with a hyperenhancing soft tissue mass that is compatible with extensive intraventricular metastatic melanoma

**FIGURE 2:**
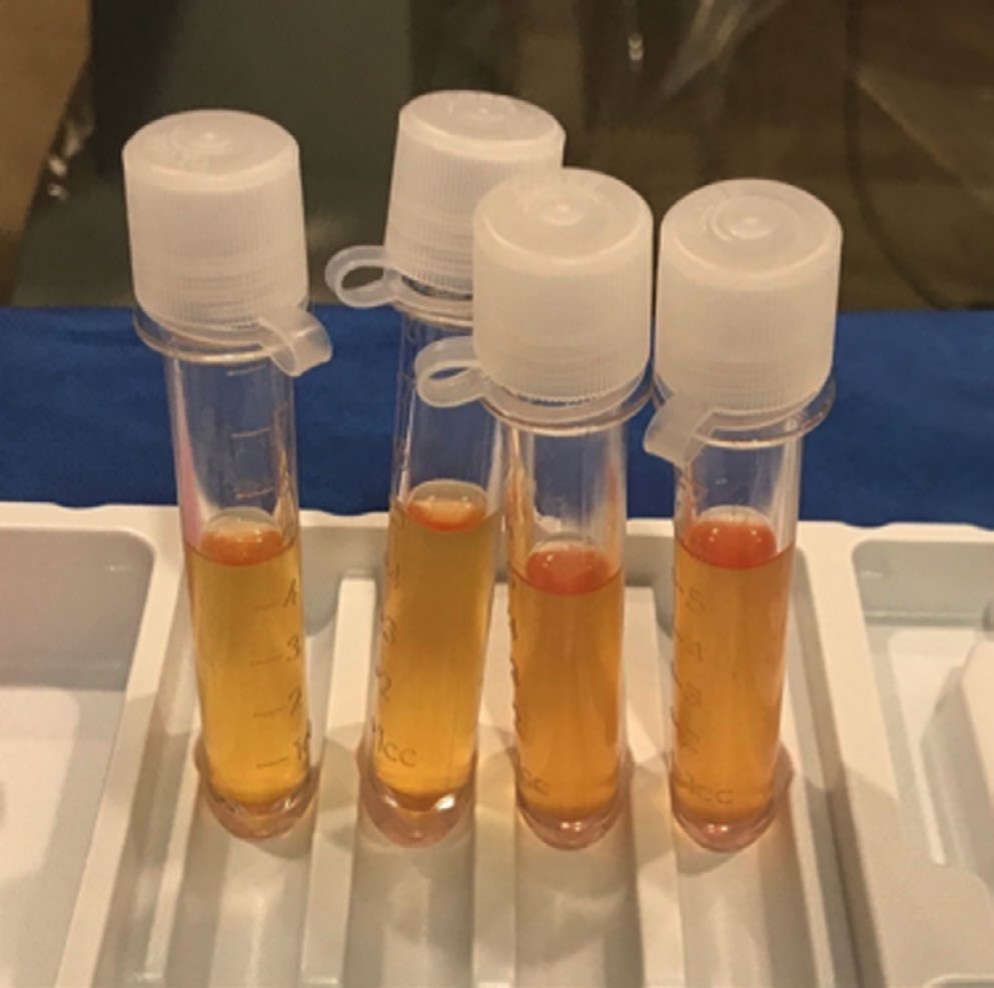
Cerebrospinal fluid from the patient was pigmented. Cytological analysis was consistent with an inflammatory exudative process and did not identify tumor cells in the specimen

**FIGURE 3:**
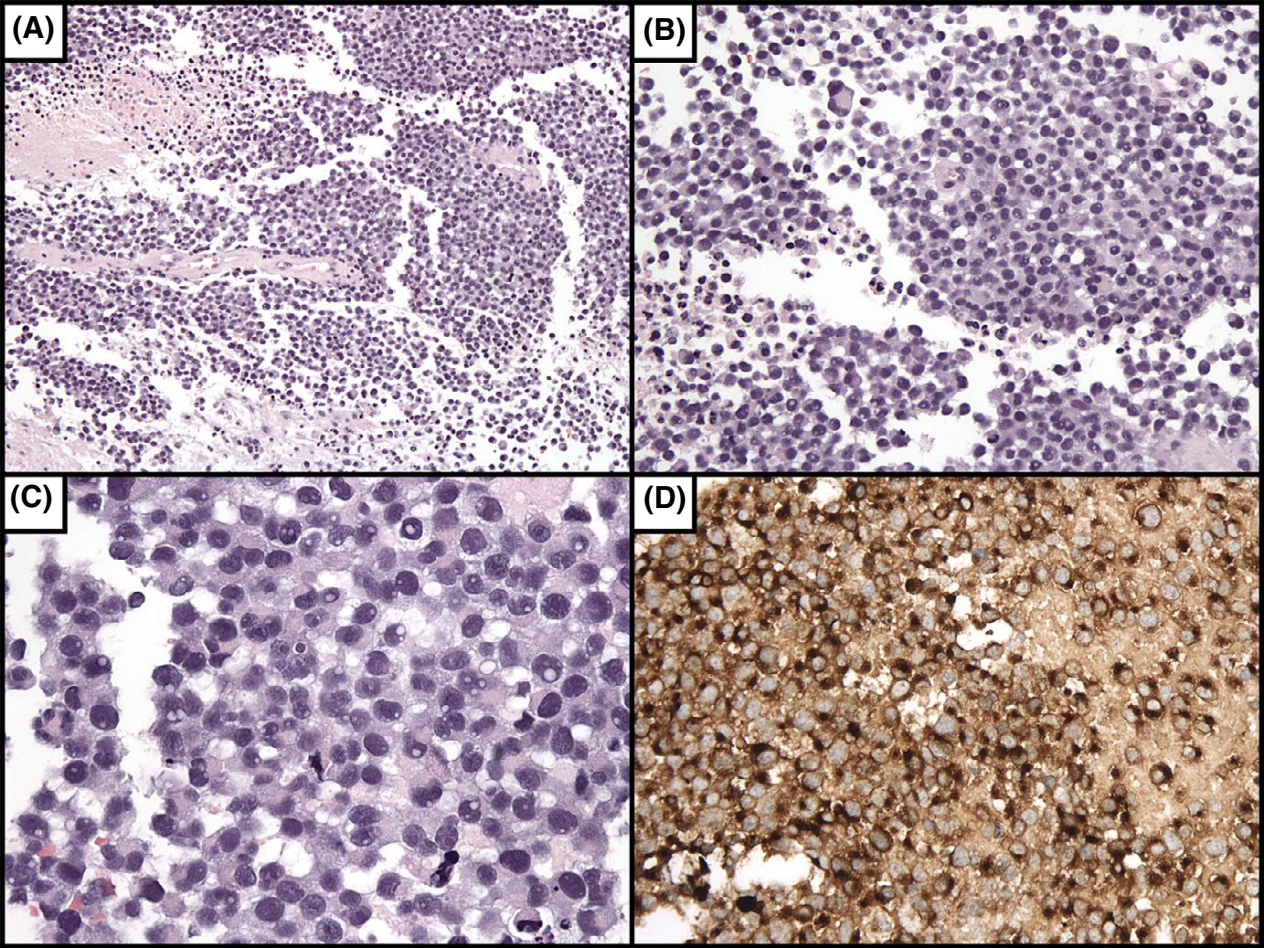
Histopathology of intraventricular biopsy specimen. A, 100x H&E staining demonstrating discohesive malignant cells with necrosis and no melanin present. B, 200x H&E staining highlights nuclei of malignant cells exhibiting intranuclear pseudoinclusions. C, 400x H&E showing mitotic figures and intranuclear pseudoinclusions. D, 200x anti‐Mart‐1 staining displaying tumor cells staining strongly for the melanoma marker Mart‐1

A 67‐year‐old male with history of resection of cutaneous melanoma originating on his back with inguinal node spread at the time of diagnosis (stage IIIa) presented with headaches, fatigue, unintentional weight loss, confusion, hyponatremia, and a newly diagnosed brain lesion. While on a prolonged cross‐country trip across the United States, he developed progressively worsening intermittent confusion, fatigue, daily nausea and vomiting, and occasional headaches over the course of one month, prompting his presentation to a local emergency department. Workup revealed a space‐occupying lesion involving the ventricular system, leading to his transfer to a tertiary center for further neurosurgical management. Three years prior to presentation, the patient was diagnosed with melanoma and underwent radical excision with inguinal node dissection at another institution, but he declined further systemic treatment despite positive nodal spread. Neurologic examination demonstrated an alert but disoriented patient without focal motor or sensory deficits. Imaging workup with magnetic resonance imaging (MRI) revealed a contrast‐enhancing intraventricular tumor occupying both the lateral, third, and fourth ventricles (Figure 1), associated with mild diffusion restriction (Figure 1C). This more diffuse, ependymal pattern is uncommon and unique as compared to the majority of reports of solitary intraventricular melanoma (Table 1). Further workup included fluoroscopic‐guided lumbar puncture that revealed an opening pressure of 55 cmH_2_0 and a cerebrospinal fluid profile that was as follows: lymphocytic predominant (86%) white blood cell count (WBC) of 87/cmm, a red blood cell count (RBC) of 5893/cmm (positive for xanthochromia), and protein and glucose concentrations of 524 and 20 mg/dL, respectively. Cytologic evaluation revealed rare isolated atypical cells in the hyperpigmented orange‐tinged fluid (Figure 2), and flow cytometry analysis showed that 33% of total cells were lymphocytes, 36% monocytes, and 31% granulocytes with no evidence of a monoclonal subset, consistent with an inflammatory exudative process. Over a two‐week course, the patient suffered a rapid neurologic decline leading to placement of bilateral external ventricular drains and acute institution of measures to manage increased intracranial pressure secondary to the development of acute obstructive hydrocephalus. Ultimately, a frameless needle biopsy under neuronavigation guidance was performed. In this particular case, safe surgical resection was not feasible; however, given the atypical location, a frameless needle biopsy was required to confirm the diagnosis and to perform cytologic and genetic analyses. Despite a known history of melanoma, radiographic findings were not definitive of a metastatic process and tissue was needed to inform treatment decisions such as initiation of an immunotherapy vs a targeted molecular therapy. Microscopic examination (Figure 3) demonstrated metastatic malignant melanoma characterized by large and markedly atypical amelanotic cells forming diffuse sheets with no vasocentricity surrounded by abundant hemorrhage and necrosis. Tumor cells stained positively for both S‐100 and MART‐1, and mutation analysis was positive for BRAF mutation, thus confirming the diagnosis of melanoma. Given the patient's grim prognosis after diagnosis and poor neurologic status after his rapid decline, his family elected to pursue hospice care, and palliation.

**TABLE 1:** Overview of 14 case reports of intraventricular melanoma

Author	Year	Age	Sex	Presenting history	Findings on imaging/location	Histopathology	Management	Outcome
Arbelaez et al^14^	1999	48	F	Headache with papilledema. Negative dermatologic and ophthalmologic examination for melanoma	CT: Hyperdense contrast‐enhancing mass involving the atrium of the left lateral ventricle (LV) with enlargement of the temporal horn. MRI: T1; 4 cm rounded mass in the left LV. T2; Hypointense lesion without surrounding edema/invasion. T1 + contrast; homogenously enhancing lesion. Diagnostic cerebral angiography: Avascular tumor	Hypercellular neoplasm composed of spindle and epithelioid malignant cells arranged predominantly in sheets. Focal areas of necrosis and brisk mitotic rate. Scattered neoplastic cells displayed dusky brown intracytoplasmic pigment. Strong immunoreactivity to HMB‐45, uniformly nonreactive to cytokeratin, glial fibrillary acid protein, and leukocyte common antigen stains	Partial resection of mass via left temporal approach. Received further therapy at another institution	Stable at 3 mo post‐op follow‐up
Lana‐Peixot et al^15^	1977	33	M	Severe headache, nausea, vomiting, left homonymous hemianopsia (left visual field) and left lower extremity weakness/numbness. Normal optic fundi. Later with myelopathy due to spinal cord and cauda equina involvement	CT: Hyperdense lesion involving the right lateral ventricle and posterior parietal lobe with surrounding edema. No imaging of the spinal axis was reported	LM: Normal choroid plexus with calcospherites and psammoma bodies. Hyperplastic choroid plexus epithelium in papillary fronds with moderate pleomorphism and hyperchromatism. Irregular and ill‐defined papillary structures containing markedly anaplastic and heavily pigmented cells	Steroids, decompressive lumbar laminectomy (for decompression of spinal cord and cauda equina),chemoradiation	Progressive worsening and ultimately died 11 mo after initial presentation
Beatty^16^	1972	8	M	Headache, lethargy, poor concentration, papilledema, and nystagmus. Negative dermatologic and ophthalmologic examination for melanoma	Ventriculogram: IV location of mass	LM: papillary arrangement. Fine pigment granules in the proliferated epithelial cells	Transcortical removal of tumor. Radiation	Required subsequent operations and radiation. Ultimately died 13 mo after initial presentatio
Khoshyomn et al^6^	2002	73	M	Headache, nausea, vomiting. Remote history of excision of a cutaneous malignant melanoma with negative axillary lymph node dissection	MRI: Multiple enhancing intraventricular nodules involving the lateral, third, and fourth ventricles	Strong immunoreactivity to HMB45	Endoscopic exploration and biopsy via right frontal burr hole. Radiation and IV/IT chemotherapy	No follow‐up reported
Escott et al^17^	2001	32	M	Headache	CT: Single metastasis involving the left trigone of the lateral ventricle. Liver metastases and innumerable pulmonary nodules	No histopathology reported	Gamma knife radiosurgery	Lesion decreased in size. No further follow‐up reported.
Escott et al^17^		31	F	History of malignant melanoma of the right thigh two years prior	MRI: Axial T1 + contrast with subtle foci of enhancement along the margins of the lateral ventricles, with subsequent Axial T1 + contrast showing much larger subependymal nodules along the superior aspects of the bodies of the lateral ventricles	No histopathology reported	No treatment reporte	No follow‐up reported
Bojsen‐Møller et al^18^	1977	19	M	Headache, diplopia, papilledema, decreased visual acuity, signs meningeal irritation. Negative ophthalmologic examination for melanoma	Pneumoencephalography: Tumor obstructing the foramen of Monro causing hydrocephalus	Melanin on autopsy specimen	No treatment reported	Died 2 y after initial diagnosis
Enzmann et al^19^	1978	Unk	Unk	No symptoms reported.	CT: Lesion involving the right occipital horn with diffuse intraventricular spread. Enhancement of both frontal horns, greater on the right	No histopathology reported	No treatment reporte	No follow‐up reported
Tien^20^	1991	63	M	No symptoms reported.	MRI: T1‐weighted sequence with a 2.5‐cm round mass filling the left frontal horn near the foremen of Monro. Hyperintense rim most likely representing methemoglobin from hemorrhage, and a hypointense center	No histopathology reported	No treatment reported	No follow‐up reported
Keraliya et al^10^	2015	54	F	No symptoms reported.	MRI: T2‐ weighted sequence demonstrated an intraventricular mass involving the left lateral ventricle. Avid contrast enhancement on T1 + contrast	No histopathology reported	No treatment reported	No follow‐up reported
Feletti et al^11^	2013	60	F	Headache, vomiting, confusion. History of resection of left knee melanoma and subsequent regional lymph node dissection for node spread	MRI: T1 + contrast sequence with a hemorrhagic, partially intraventricular lesion in the left occipital horn with fluid levels	Histology of the lesion showed sheets of neoplastic cells with large eosinophilic cytoplasm and atypical nuclei with large nucleoli, along with hemorrhagic areas. High mitotic activity. Strongly positive immunoreactivity for melanocytic marker HMB45 (+++), Negative immunoreaction for cytokeratin marker MNF116	Resection via an interhemispheric transcallosal approach with adjuvant radiation	No recurrence at 8 mo
Cipri et al^21^	2009	22	M	Intermittent headache, vomiting and disequilibrium. No history of cutaneous lesions. Had inguinal, bronchopulmonary and suprarenal lymph node spread	CT: biventricular hydrocephalus secondary to 2‐cm lesion in superior aspect of third ventricle mimicking a colloid cyst MRI: Contrast‐enhancing lesion arising from the lower part of the septum pellucidum	IHC staining of bronchoscopy, neuroendoscopic and lymph node samples with strongly positive Melan and HMB45 and weakly positive S‐100	Neuroendoscopic septostomy, ventriculoperitoneal shunt. Stereotactic radiosurgery and chemotherapy.	No follow‐up reported
Gaab et al^22^	1999	62	M	Drowsiness and confusion	CT and/or MRI: Third ventricle and midbrain involvement with hydrocephalus	No histopathology reported	Third ventriculostomy	Died 5 d post‐op
Dasgupta et al^23^	1964	Unk	Unk	No symptoms reported	Choroid plexus	No histopathology reported	No treatment reported	No follow‐up reported

Abbreviations: CT, computed tomography; IHC, immunohistochemistry; IT, intrathecal; IV, intraventricular; LM, light microscopy; MRI, magnetic resonance imaging.

## 3 DISCUSSION

Central nervous system metastasis is frequent in melanoma, with a lifetime prevalence of up to 46% survivors of the primary disease.^8^ In 14 cases of IVT metastatic melanoma reported in the literature (Table 1), patients had a median age of 42.1 years (range 8‐73 years). Race was not identified in any of the reported cases. Risk factors associated with CNS involvement in melanoma include male gender, mucosal or head and neck primaries, thick or ulcerated neoplasms, and acral lentiginous or nodal disease. Time from primary disease to diagnosis of IVT metastases is reportedly 3.8 years,^9^ but presentation up to 36 years after excision of cutaneous primary melanoma has been reported.^6^ Although our patient had a history of cutaneous melanoma and excision, a significant number of cases have no history of a cutaneous primary (Table 1). Primary CNS melanoma represents a diagnosis of exclusion after ruling out primary sites involving the skin, eyes, and mucosal surfaces, which are frequent sources of CNS metastasis.^10^ The most commonly reported site of origin for IVT melanoma includes arrested melanocytic cells in the choroid plexus, which may represent the separate diagnostic entity of CNS melanocytoma.^10^, ^11^ The most common location of involvement within the ventricular system is the lateral ventricles.^11^ Headache, vomiting, and nausea are the most common presenting symptoms (Table 1). Additionally, papilledema is almost universally discovered on fundoscopy, with elevated opening pressures, and hemorrhage on reported CSF studies. In this patient, the hyponatremia may represent a syndrome of inappropriate antidiuretic hormone (SIADH) in the setting of malignancy or it may be a representation of unknown adrenal or pituitary metastases. Common CT findings include hyperdense contrast‐enhancing masses, usually in the lateral ventricles (Table 1). MRI typically shows T1 enhancement (Table 1). Obstructive hydrocephalus is also frequently seen on imaging. The diagnosis can be by histopathology of the endoscopically resected specimen. Common histopathologic findings include epithelioid cells arranged in sheets with significant intracytoplasmic pigment and significant immunoreactivity to HMB‐45 staining (Table 1). The major differential diagnoses of CNS melanoma include melanotic tumors such as melanotic schwannoma, pigmented choroidal papilloma, melanotic meningioma, melanotic paraganglioma, and benign melanocytoma.^12^ Management typically involves endoscopic resection along with chemotherapy and radiation; however, outcomes were dismal in all cases that reported survival (Table 1), indicating that new therapeutic options are needed for this rare pattern of metastatic melanoma. Tawbi et al. support the exploration of immunotherapeutic options given the recent success of such strategies (eg, combined nivolumab and ipilimumab) in treating intraparenchymal melanoma metastases.^13^

## 4 CONCLUSION

Intraventricular metastasis of melanoma is a rare and highly malignant disease entity that is poorly understood due to the paucity of cases. Surgical resection is the main treatment method, with no clear guidelines on adjuvant chemotherapy or radiation. Careful pathological examination is required to confirm the diagnosis. Complications typically include elevated intracranial pressures, frequently requiring CSF diversion. Outcomes are poor, and new treatment approaches are needed.

## CONFLICT OF INTEREST

JDB has positions/equity in CITC Ltd. and Avidea Technologies and is a member of the board of scientific advisors for POCKiT Diagnostics. All other authors declare that they have no competing conflicts of interest.

## AUTHOR CONTRIBUTIONS

All authors participated in the clinical care of the patient and/or the drafting/revising of the manuscript. JDB: involved in concept and design; acquisition of data; and drafting/revision of the manuscript. GC: involved in concept and design; acquisition of data; and drafting/revision of the manuscript. GE and BF: performed analysis and interpretation of data; and drafting/revision of the manuscript. NM involved in concept and design; and drafting/revision of the manuscript. SK and MC drafted/revised the manuscript. TJA drafted/revised the manuscript; and involved in submission of manuscript. HS, JH, GKF, and MRH involved in concept and design; and supervised the work.
